# Brazilian researchers meet online to discuss social distancing: Results from the first symposium of the group of studies and research on psychology and cognition (GEPESPSI)

**DOI:** 10.1192/j.eurpsy.2021.691

**Published:** 2021-08-13

**Authors:** C. Varanda, A. Macedo Filho

**Affiliations:** 1 Institute Of Human Sciences - Faculty Of Psychology, Universidade Paulista, SANTOS, Brazil; 2 Human Sciences, Universidade Paulista, Santos, Brazil

**Keywords:** social distancing, virtual symposium, Psychology

## Abstract

**Introduction:**

The Group of Studies and Research on Psychology and Cognition (GEPESPSI) in Brazil has developed important academic and clinical actions on mental health in contexts of difficult psychological handling.

**Objectives:**

GEPESPSI organized a symposium to discuss the psychological effects of isolation due to the strict measures of social distancing.

**Methods:**

11 psychologists and one speech language pathologist discussed possible contributions to face social distancing in their specific fields of expertise in a virtual symposium of two days. The themes were: university teaching; support to the development of social and emotional competencies among children; the threat of the death of dreams in a phenomenological perspective; resilience and self-esteem; the repercussions of the lack of the school space for socialization; psychological tools for facing isolation; the challenges and perspectives of women who are victims of violence; online therapy for children; the health of workers; suicidal behavior; formulation of educational policies for remote learning and family mental health.

**Results:**

1094 people were enrolled with an average of 400 people participating in each period. 91,1% of which were graduate students of a university. 55,7% were students of Psychology, 12,8% of Pedagogy, 6% of Physical Therapy among other courses. 39,8% of them were from the city of Santos, the others were from different regions of the country.

**Conclusions:**

The feedback given by the participants was positive and involved gains such as sharing experience and knowledge but mainly creating connections to exchange psychological tools as a way of facing the difficulties of social distancing among researchers and graduate students.

